# Supplementary material for: Structure-function analyses reveal key molecular determinants of HIV-1 CRF01_AE resistance to the entry inhibitor temsavir
Source: Nat Commun. 2023 Oct 23;14:6710. doi: 10.1038/s41467-023-42500-2 (PMC10593844; doi:10.1038/s41467-023-42500-2)
Supplement: Supplementary file 1 — Supplementary Information [file 41467_2023_42500_MOESM1_ESM.pdf]

Supplementary Information for

Structure-function Analyses Reveal Key Molecular Determinants of HIV-1 CRF01_AE Resistance to the Entry Inhibitor temsavir

Jérémie Prévost[1,2,#], Yaozong Chen[3,#], Fei Zhou[4], William D. Tolbert[3], Romain Gasser[1,2], Halima Medjahed[1], Manon Nayrac[1,2], Dung N. Nguyen[3], Suneetha Gottumukkala[3], Ann J. Hessell[5], Venigalla B. Rao[6], Edwin Pozharski[7], Rick K. Huang[8], Doreen Matthies[4], Andrés Finzi[1,2,*], Marzena Pazgier[3,*,&]

[1]Centre de Recherche du CHUM, Montreal, QC, Canada;

[2]Département de Microbiologie, Infectiologie et Immunologie, Université de Montréal, Montreal, QC, Canada;

[3]Infectious Disease Division, Department of Medicine, Uniformed Services University of the Health Sciences, Bethesda, MD, USA;

[4]Unit on Structural Biology, Division of Basic and Translational Biophysics, *Eunice Kennedy Shriver* National Institute of Child Health and Human Development, NIH, Bethesda, MD, USA;

[5]Oregon National Primate Research Center, Oregon Health and Science University, Beaverton, OR, USA.

[6]Department of Biology, the Catholic University of America, Washington, DC, USA;

[7]Institute for Bioscience and Biotechnology Research, Rockville, MD 20850, USA; Department of Biochemistry and Molecular Biology, University of Maryland School of Medicine, Baltimore, MD, USA

[8]Laboratory of Cell Biology, National Cancer Institute, NIH, Bethesda, USA;

[#]These authors contributed equally

*Correspondence:

Andrés Finzi: andres.finzi@umontreal.ca

Marzena Pazgier: marzena.pazgier@usuhs.edu

[&]Lead Contact

This PDF files includes:

Table. S1-S3

Fig. S1 to S10

Table S1. Cryo-EM data collection and refinement statistics.

	CRF01_AE_ T/F100 SOSIP.664 in complex with 10-1074 and 8ANC195			BG505 SOSIP.664 in complex with 10-1074 and 8ANC195
	**Wild type apo**	**LMHS apo**	**LMHS/temsavir bound**	**temsavir bound**
EMDB	EMD-27596	EMD-29783	EMD-27103	
PDB	8DOK	8G6U	8CZZ	
**Data Collection**				
Microscope	G1 TITAN KRIOS	G1 TITAN KRIOS	Glacios	Glacios
Voltage (kV)	300	300	200	200
Detector	Gatan K3	Gatan K3	Gatan K3	Gatan K3
Pixel Size (Å/px)	0.83	0.83	0.8893	0.888
Dose rate (e^-/px/s)_	15	15	15.4	13.8
Total exposure dose (e^-/Å^2)	54.4	54.4	58.1	43.8
Dose per frame (e^-/Å^2)	1.09	1.09	1.29	1.22
Total exposure time (s)	2.5	2.5	3.0	2.5
Exposure time per frame (s)	0.05	0.05	0.07	0.07
Defocus Range (µm)	0.5-2.7	0.5-2.7	0.5-2.7	0.5-2.7
Magnification (calibrated)	105,000x (60,241x)	105,000x (60,241x)	45,000x (56,224)	45,000 (56,306)
**Reconstruction**				
Software	CryoSPARC	CisTEM	CryoSPARC	CryoSPARC
Micrograph collected (selected)	4087 (3932)	7594 (7580)	1484 (1449)	4238 (3707)
Number of final particles	476,274	549,061	234,547	319,575
Symmetry	C3	C3	C3	C3
Box size (px)	324	486	324	360
Resolution (Å) (FSC 0.143)	3.20	3.16	3.14	2.96
B-factor	85.7	86.7	91.9	116.9
**Refinement (Phenix) & validation**				
Protein residues	3091	3114	3114	3744
Chimera CC	0.83	0.81	0.77	0.82
EMRinger Score				
RMSD Bond lengths (Å)	0.006	0.005	0.008	0.004
RMSD Bond angles (˚)	1.049	1.044	1.395	0.662
Molprobity score	1.87	2.61	2.13	3.24
Clash score	12.34	14.33	12.40	31.50
Rotamer outliers (%)	0.71	3.79	2.12	10.14
**Ramachandran**				
Favored (%)	96.07	91.45	95.96	91.80
Disallowed (%)	0.17	0.98	0.13	0.62

Table S2. Interfaces of 8ANC197 and 10-1074 bound to CRF01_AE_ T/F100 SOSIP.664 wild type and its LMHS mutant bound or unbound to temsavir and bound to BG505 SOSIP.664 bound to temsavir. The Fab-Env buried surface area (BSA) for each antibody and each complex structure was calculated using the EBI PISA server (http://www.ebi.ac.uk/msd-srv/prot_int/cgi-bin/piserver). BSA values for each protomer (an average of three protomers) are shown for both the protein component and the glycan (in parenthesis).

		**CRF01_AE_ T/F100 SOSIP.664**						**BG505 SOSIP.664**	
		**Wild type** (apo) **8ANC195**	**LMHS** (apo) **8ANC195**	**LMHS/ temsavir 8ANC195**	**Wild type** (apo) **10-1074**	**LMHS** (apo) **10-1074**	**LMHS/ temsavir 10-1074**	**Wild type/ temsavir 8ANC195**	**Wild type/ temsavir 10-1074**
**Buried Surface Area, Å^2**	**SOSIP total**	**1306 (1918)**	**1315 (1844)**	**1183 (1774)**	**442 (524)**	**403 (728)**	**401 (459)**	**1193 (520)**	**555 (519)**
	**Combined (protein+glycan)**	**3227**	**3159**	**2957**	**966**	**1131**	**860**	**1713**	**1074**
	gp120	754 (1458)	743 (1303)	699 (1340)	442 (524)	403 (728)	401 (459)	648 (250)	555 (519)
	gp41	552 (460)	572 (541)	484 (434)	0 (0)	0 (0)	0 (0)	545 (270)	0 (0)
	**Heavy chain total**	**1040 (1033)**	**1046 (1027)**	**980 (997)**	**279 (290)**	**273 (318)**	**257 (304)**	**931 (218)**	**301 (232)**
	**Combined (protein+glycan)**	**2073**	**2073**	**1977**	**569**	**591**	**561**	**1149**	**533**
	FWR	317 (762)	306 (724)	274 (728)	0 (0)	0 (2)	0 (0)	255 (207)	0 (0)
	CDR H1	90 (0)	99 (0)	88 (0)	0 (0)	0 (0)	0 (0)	94 (0)	0 (0)
	CDR H2	86 (240)	79 (256)	92 (239)	0 (0)	0 (0)	0 (0)	86 (0)	0 (0)
	CDR H3	547 (31)	562 (47)	526 (30)	279 (290)	273 (318)	257 (304)	496 (11)	301 (232)
	**Light chain total**	**304 (314)**	**319 (369)**	**278 (324)**	**195 (118)**	**157 (292)**	**182 (77)**	**283 (175)**	**286 (232)**
	**Combined (protein+glycan)**	**618**	**688**	**602**	**313**	**449**	**259**	**458**	**518**
	FWR	6 (51)	13 (75)	0 (54)	42 (80)	20 (119)	41 (58)	0 (27)	51 (103)
	CDR L1	226 (75)	246 (71)	213 (79)	34 (1)	41 (6)	28 (0)	219 (21)	38 (19)
	CDR L2	65 (188)	60 (223)	61 (190)	0 (35)	0 (167)	0 (19)	58 (127)	0 (86)
	CDR L3	7 (0)	0 (0)	4 (1)	119 (2)	96 (0)	113 (0)	6 (0)	197 (24)
	**Fab total**	**1344 (1347)**	**1365 (1396)**	**1258 (1321)**	**474 (408)**	**430 (610)**	**439 (381)**	**1214 (393)**	**587 (464)**
	**Interface Total**	**2650 (3265)**	**2680 (3240)**	**2441 (3095)**	**916 (932)**	**833 (1338)**	**840 (840)**	**2407 (913)**	**1142 (983)**
	**Combined (protein+glycan)**	**5915**	**5920**	**5536**	**1848**	**2171**	**1680**	**3320**	**2125**

Table S3. Primer sequences for site-directed mutagenesis

Mutant	Forward primer	Reverse primer
T/F100.R6.SOSIP.664 H61Y	5’-GCGACGCCAAGGCCTACGAGACAGAGG-3’	5’-CCTCTGTCTCGTAGGCCTTGGCGTCGC-3’
T/F100.R6.SOSIP.664 Q105H V108I	5’- GAACAGATGCACGAAGATATCATCAGCCTG-3’	5’-CAGGCTGATGATATCTTCGTGCATCTGTTC-3’
T/F100.R6.SOSIP.664 N474D I475M K476R	5’-CCCGGCGGAGGCGATATGAGGGACAACTGGCG-3’	5’-CGCCAGTTGTCCCTCATATCGCCTCCGCCGGG-3’
T/F100.R6.SOSIP.664 H375S	5’-GATCACCATGCACAGCTTCAACTGTCGG-3’	5’-CCGACAGTTGAAGCTGTGCATGGTGATC-3’

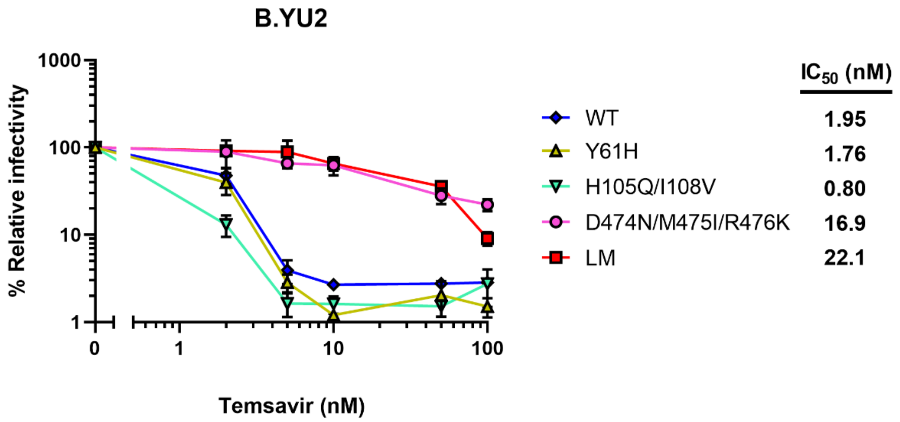

Fig. S1. Impact of individual gp120 inner domain Layer residues on temsavir neutralization sensitivity. Recombinant HIV-1 pseudoviruses expressing luciferase and bearing WT or mutated Env from clade B YU2 were used to infect Cf2Th-CD4/CCR5 cells in the presence of increasing concentrations of temsavir. Infectivity at each dilution of the compound tested is shown as the percentage of infection without the compound for each particular mutant. Triplicate samples were analyzed in each experiment. Data shown are the means of results obtained in n=3 independent experiments. The error bars represent the standard deviations. Neutralization half maximal inhibitory concentration (IC_{50}) were calculated by non-linear regression using the Graphpad Prism software. Source data are provided as a Source Data file.

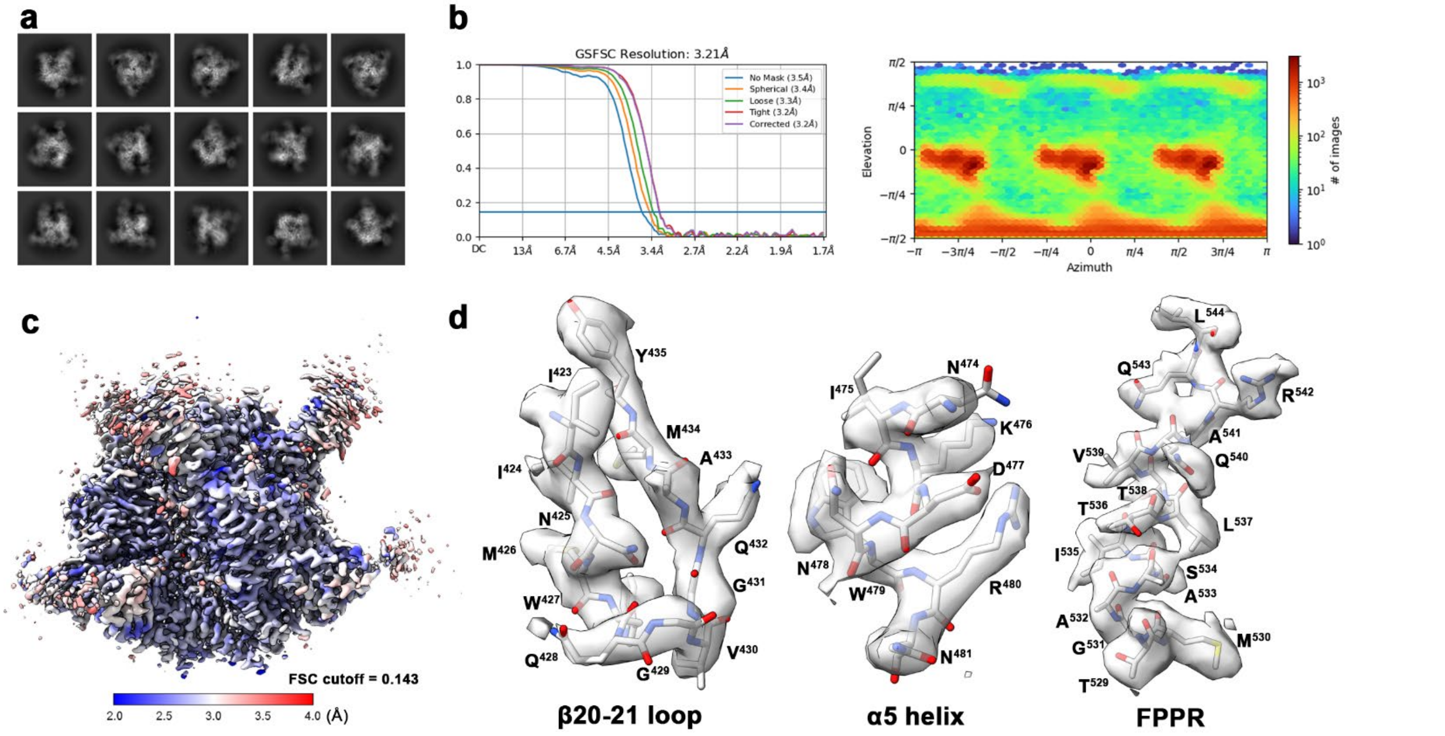

Fig. S2. Cryo-EM structure of CRF01_AE_ T/F100 SOSIP.664 wild type with two chaperone Fabs 10-1074 and 8ANC195. (**a**) Selected 2D classes for *ab initio* map reconstruction. (**b**) The Fourier shell correlation curves indicate the overall resolution (FSC cutoff 0.143) using non-uniform refinement and the direction distribution plot of all particles used in the final refinement. (**c**) Local resolution estimation. (**d**) Density and corresponding model from portions of the β20-21 loop, the α5 helix, and the Fusion Peptide Proximal Region (FPPR).

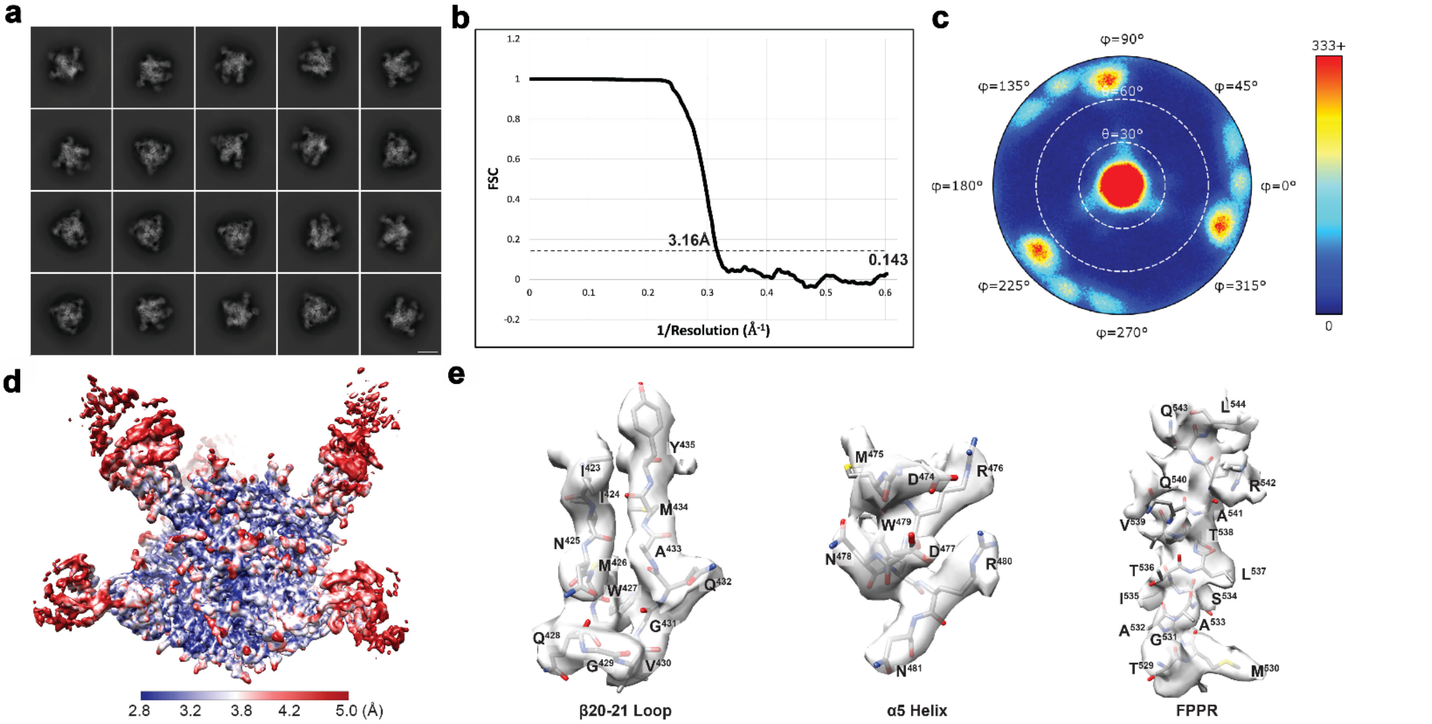

Fig. S3. Cryo-EM structure of CRF01_AE_ T/F100 LMHS SOSIP.664 in complex with chaperone Fabs 10-1074 and 8ANC195. (**a**) Selected 2D classes for *ab initio* map reconstruction. Scale bar is 10nm. (**b**), (**c**) The Fourier shell correlation curves with spherical mask indicate the overall resolution (FSC cutoff 0.143) using cisTEM auto-refinement and the direction distribution plot of all particles used in the final refinement. (**d**) Local resolution estimation. (**e**) Density and corresponding model from portions of the β20-21 loop, the α5 helix, and the Fusion Peptide Proximal Region (FPPR).

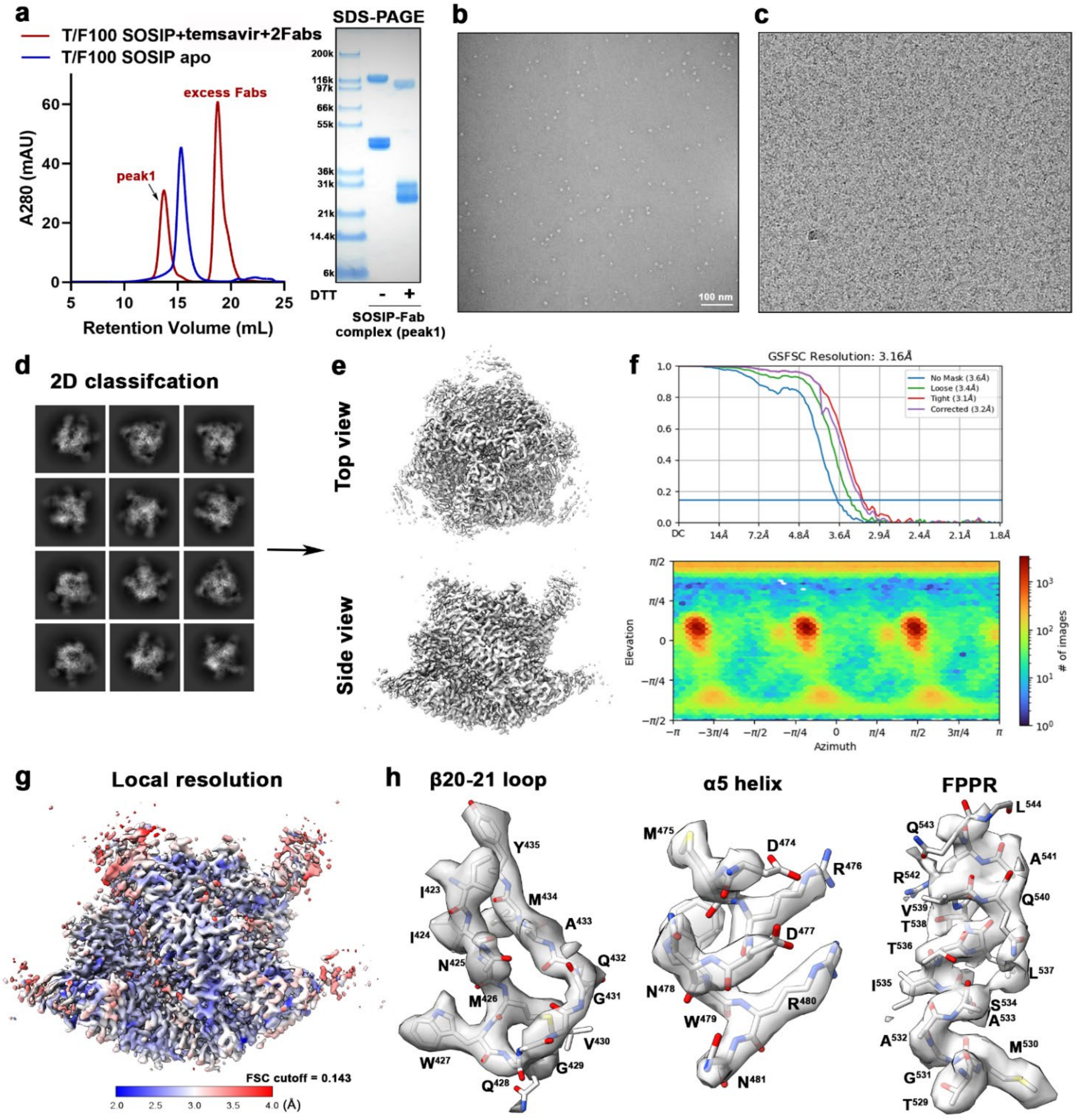

Fig. S4. Cryo-EM structure of CRF01_AE_ T/F100 LMHS SOSIP.664 mutant in complex with temsavir and two chaperone Fabs 10-1074 and 8ANC195. (**a**) Cryo-EM sample preparation. Size-exclusion chromatogram (SEC) of the purified SOSIP.664 trimer (blue) and its complex with 2 Fabs and temsavir (red) on a Superose-6 increase 300/10 GL column. The SEC peak-shifted and SDS-PAGE validated the Fab-spike complex formation. (**b**) Representative negative-stained EM image of the purified CRF01_AE_ T/F100 LMHS SOSIP.664-Fabs-temsavir complex (using JEOL-JEM1011 transmission electron microscope, complex concentration at 1mg/ml). (**c**) Representative raw cryo-EM micrograph. (**d**) Selected 2D classes for *ab initio* map reconstruction. (**e**) Side and top views of the final cryo-EM density map imposed with C3 symmetry. (**f**) The Fourier shell correlation curves indicate the overall resolution (FSC cutoff 0.143) using non-uniform refinement and the direction distribution plot of all particles used in the final refinement. (**g**) Local resolution estimation. (**h**) Density and corresponding model from portions of the β20-21 loop, the α5 helix, and the FPPR.

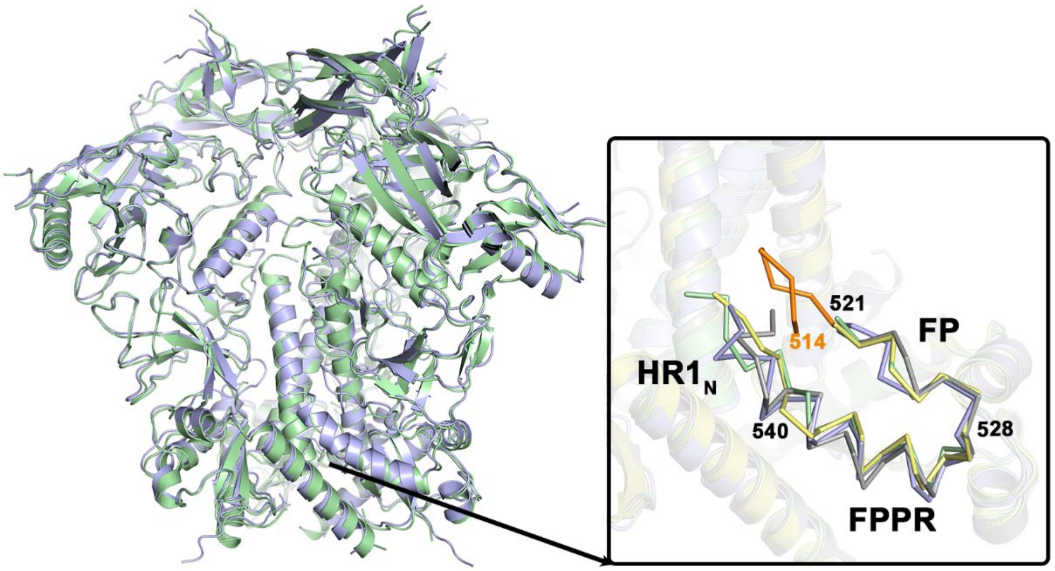

Fig. S5. Structural alignment of CRF01_AE_ T/F100 SOSIP.664 trimers. Structures of CRF01_AE_ T/F100 SOSIP.664 from this study (green) and by Ananthaswamy et al.[1], PDB: 6NQD (blue) were superimposed based on the gp41 trimer. The blow-up shows details in the conformation of the gp41 region around the fusion peptide (FP) for both CRF01_AE_ T/F100 SOSIP.664 wild type structures, LMHS apo (yellow) and LMHS-temsavir complex (gray) for the fusion peptide (FP, residues 514-527), the fusion peptide proximal region (FPPR, residues 528-540) and the N-terminal region of heptad repeat 1 ($HR1_N$, residues 541-548). The portion of the FP that is disordered and missing in CRF01_AE_ T/F100 SOSIP.664 variants from this study is colored orange. Overall structures of CRF01_AE_ T/F100 SOSIP.664 from this study and Ananthaswamy et al.[1] closely resemble each other (RMSD value for the main chain atoms of the trimer is 1.376 Å). A major difference is observed in the FP region, with the HP region spanning residues 514-521, disordered and not resolved in any of the structures of the CRF01_AE_ T/F100 SOSIP.664 variants from this study.

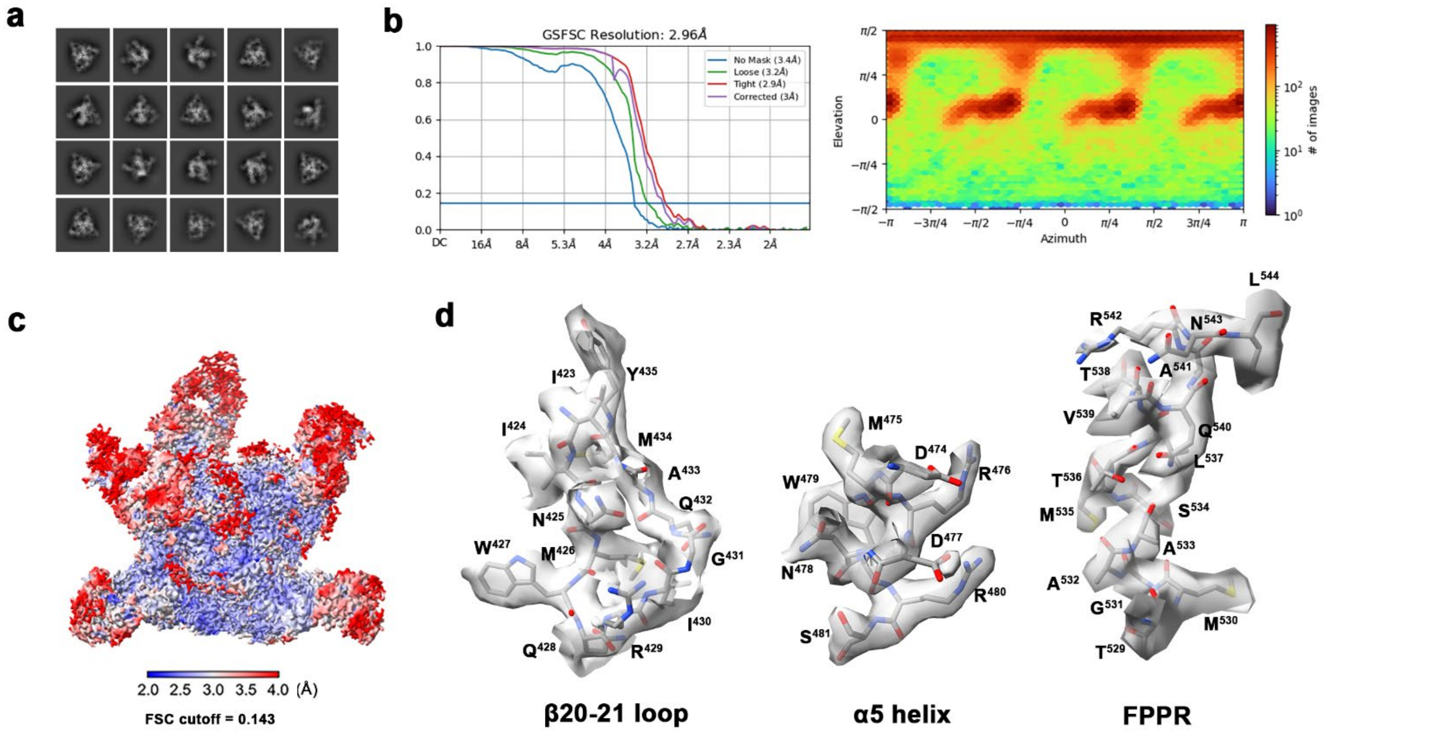

Fig. S6. Cryo-EM structure of BG505 SOSIP.664 in complex with temsavir and chaperone Fabs 10-1074 and 8ANC195. (**a**) Selected 2D classes for ab initio map reconstruction. (**b**) The Fourier shell correlation curves indicate the overall resolution (FSC cutoff 0.143) using non-uniform refinement and the direction distribution plot of all particles used in the final refinement. (**c**) Local resolution estimation. (**d**) Density and corresponding model from portions of the β20-21 loop, the α5 helix, and the Fusion Peptide Proximal Region (FPPR).

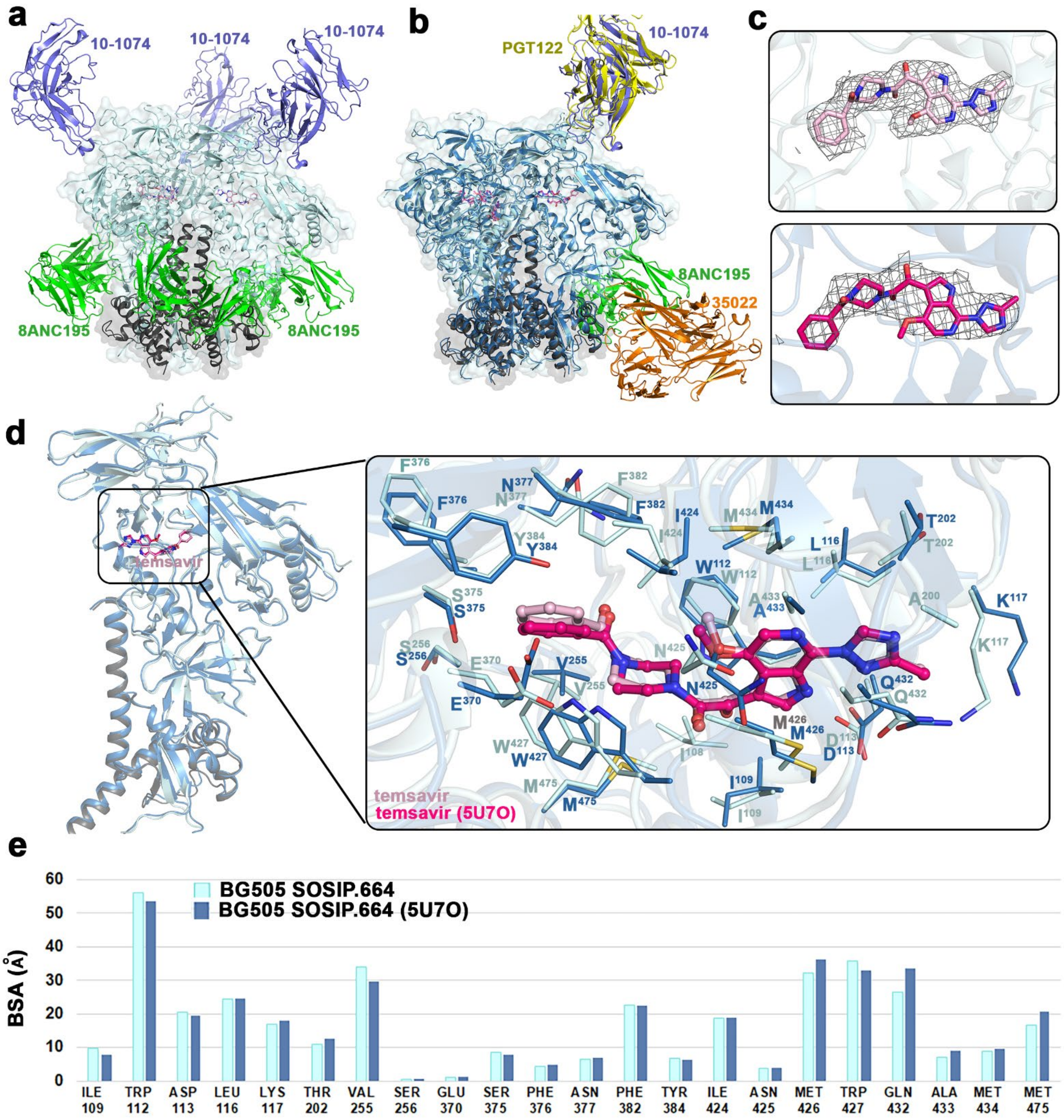

Fig. S7. Structure of temsavir-BG505 SOSIP.664 complex. (**a**) Overall Cryo-EM structure of temsavir- BG505. SOSIP.664 complex with chaperone Fabs of 10-1074 and 8ANC196 (cyan/grey for gp120/gp41 subunits and light pink for temsavir). The variable parts of chaperone Fabs are shown as cartoon. (**b**) Structural alignment of temsavir-BG505. SOSIP.664 complex from this study and the X-Ray crystallographic structure of temsavir-BG505SOSIP.664 complex with chaperone Fabs of PGT121 and 35022 (PDB:5U7O), grey for Env, magenta for temsavir). (c) Experimental density maps of temsavir from the temsavir-BG505 SOSIP.664 complex determined in this study by Cryo-EM (top panel) and by X-Ray crystallography (bottom panel) (**d**) Superimposition of gp12/gp41 protomers with a blow-up view into the details of the temsavir binding pocket. The inhibitor molecules are shown in ball-and-sticks while the pocket residues are shown as lines (**e**) The residue-resolved buried-surface-area (BSA) of gp120 contributing to the temsavir-protein interface as determined by PISA. Structural alignments of the two temsavir-BG505 SOSIP.664 complex structures indicate a close similarity of the overall trimer assembly (root mean square deviation (RMSD) of 1.23Å and 1.19Å for the trimer and protomer

respectively) as well as the temsavir binding pocket. In both structures the temsavir binding pocket is formed by the same set of residues that contribute similar BSA to the pocket. A major difference is observed in the regions accommodating the phenyl ring of temsavir with noticeable displacement of the ring. In the complex from this study the phenyl ring is shifted approximately 1Å up to pack against Phe^{382} which could be in response of the slight shift in position of Trp^{427} relative to temsavir or to the different rotamer observed for the Phe^{382} phenyl ring in this structure. Most of the other side chains within the pocket are largely superimposable highlighting the similarity of the two temsavir binding pockets.

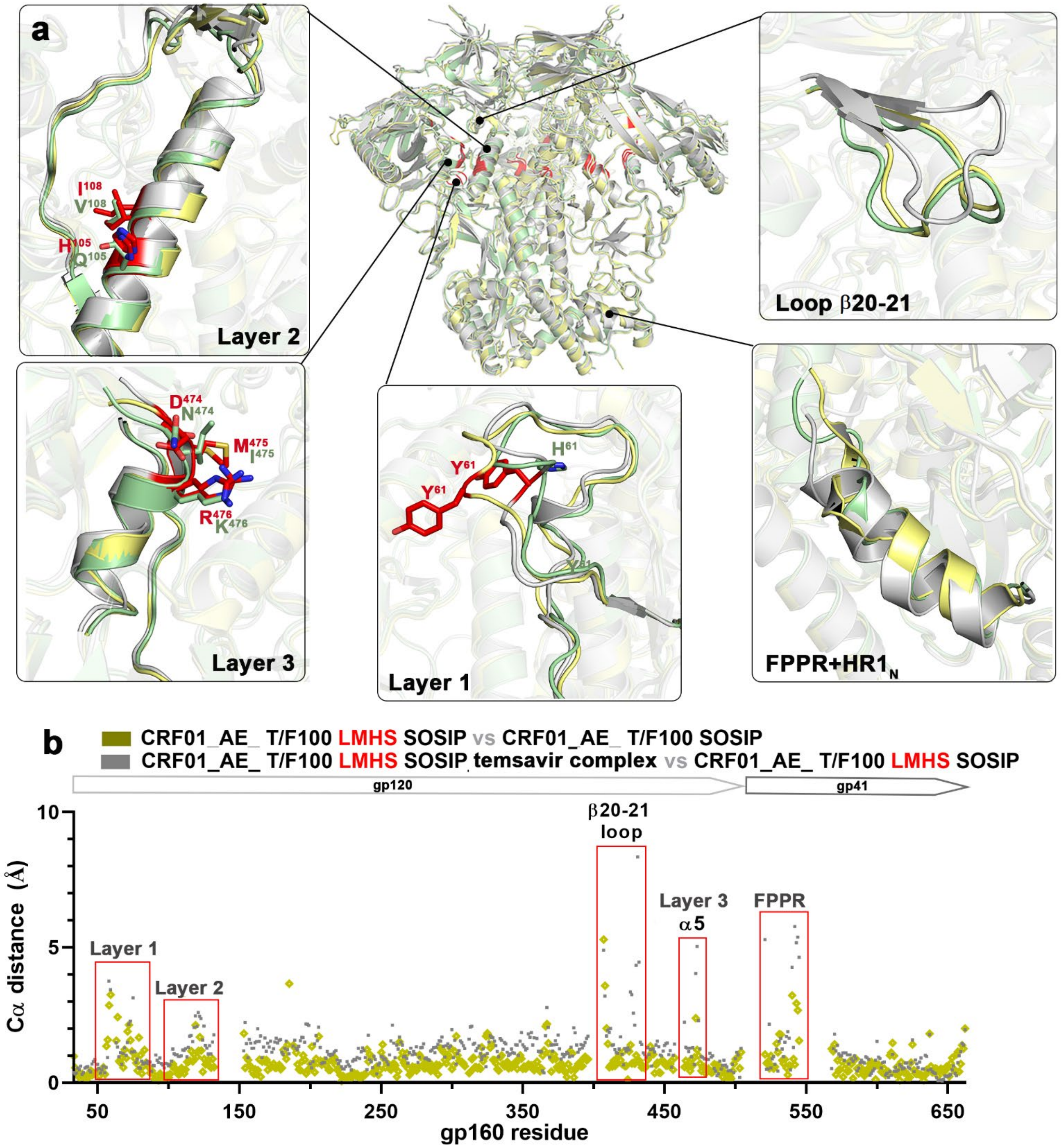

Fig. S8. Structural alignment of CRF01_AE_ T/F100 LMHS SOSIP.664 trimer variants. (**a**) Superimposition of the SOSIP trimers. Structures of CRF01_AE_ T/F100 SOSIP.664 wild type (green), CRF01_AE_ T/F100 LMHS SOSIP.664 mutant (yellow) and its complex with temsavir (gray) were aligned based on the gp41 trimer and the blow-ups views show differences in overall conformation of the backbone in regions that differed the most (Layer 1 (residues 50-82), Layer 2 (residues 97-123, 199-222), Layer 3 (residues 248-256, 473-484), the β20-21 loop (residues 424-434) and the fusion FPPR and $HR1_N$ (FPPR+$HR1_N$, (residues 528-540 and 541-548) as calculated by the root-mean-square deviation (RMSD) of Cα atoms. (**b**) Comparison of the RMSD values for Cα atoms of pairs for the CRF01_AE T/F100 SOSIP.664 wild type and its LMHS mutant and the apo LMHS mutant and its temsavir bound counterpart.

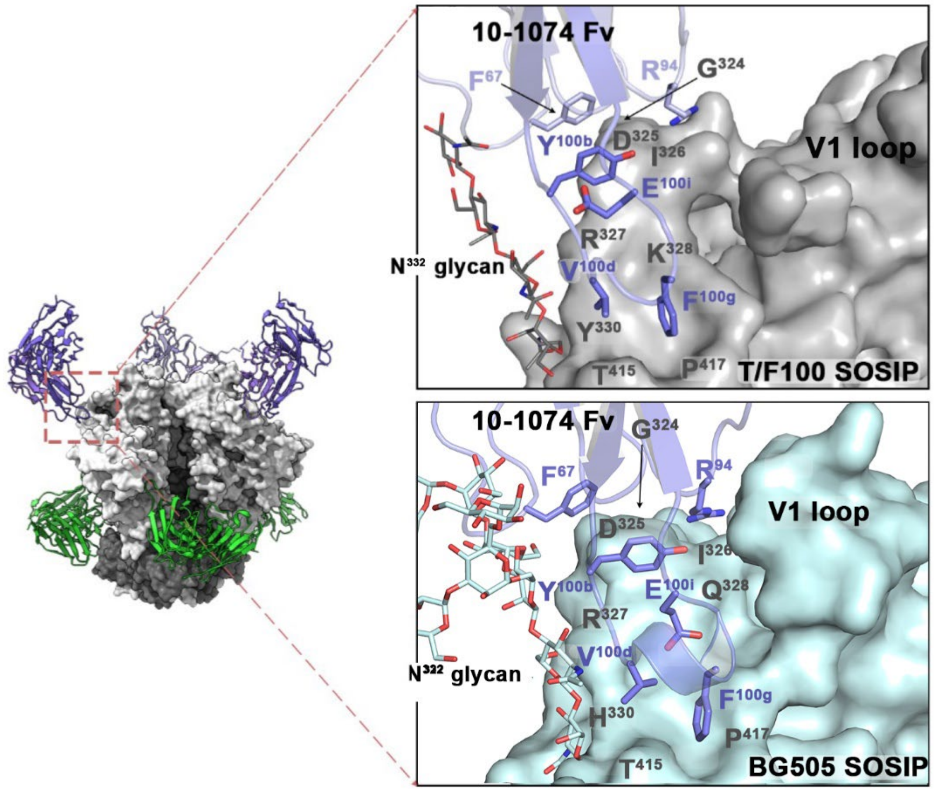

Fig. S9. Comparisons of the 10-1074 binding interfaces of CRF01_AE_ T/F100 and BG505 SOSIP.664 trimers. Structure of CRF01_AE_ T/F100 SOSIP.664 trimer in complex with Fabs of 10-1074 and 8ANC195. The blow-up views show the 10-1074 interface in CRF01_AE_ T/F100 SOSIP.664 (gray) and BG505 SOSIP.664 (cyan). The N332 glycan is shown as sticks. The glycan component of the interface is similar for the CRF01_AE_ T/F100 and BG505 SOSIP interfaces, but the protein component of the interface is slightly greater for the BG505 SOSIP structure. This is consistent with the observation that the 10-1074 interface is largely dependent upon the glycan on N332.

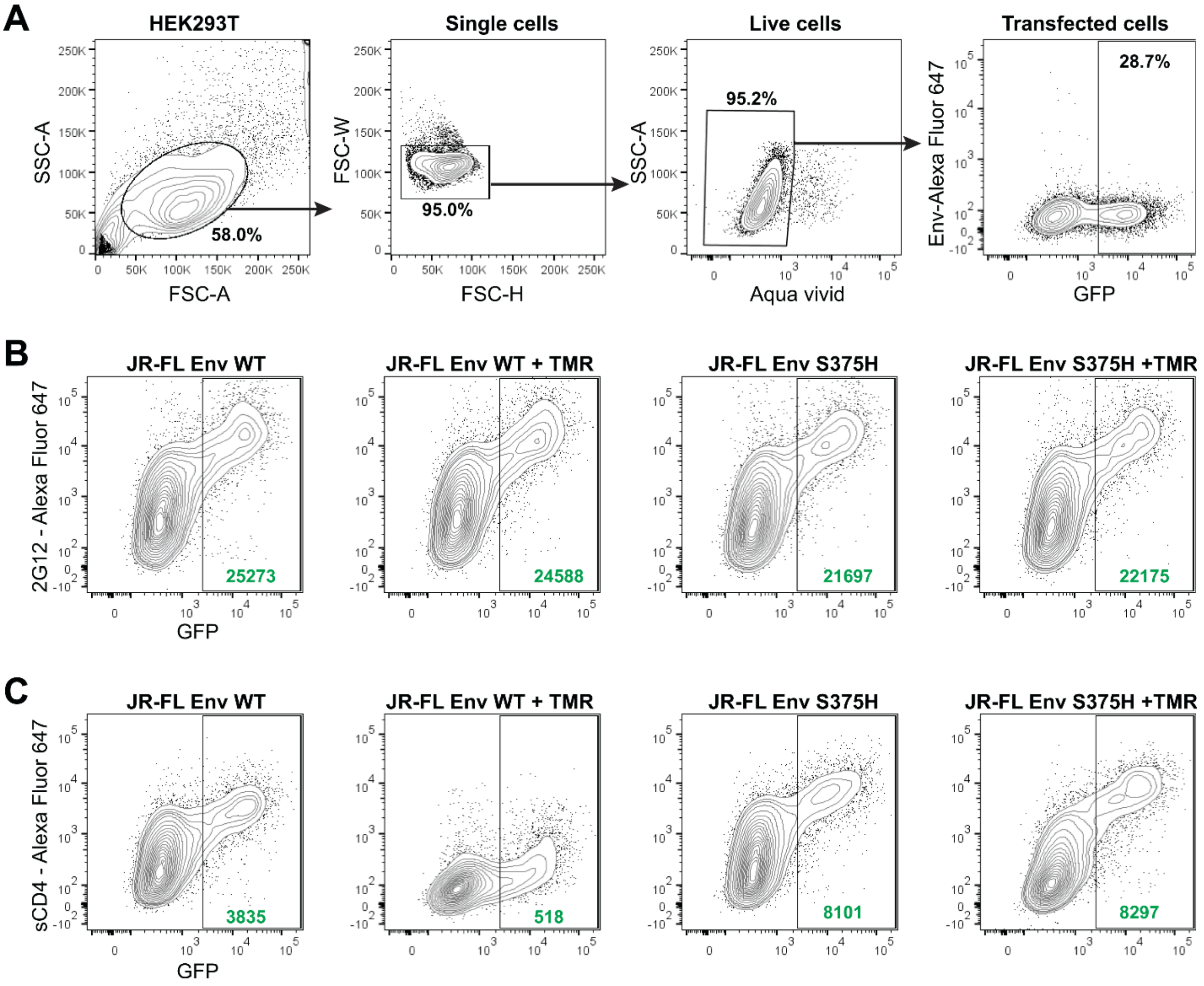

Fig. S10. Flow cytometry gating strategy for the sCD4 competition assay.
Representative flow cytometry gates to quantify the binding of soluble CD4 (sCD4) at the surface of HIV-1 Env-expressing cells. (**a**) Transfected HEK293T cells were identified according to cell morphology by light-scatter parameters, followed by exclusion of doublets cells, Aqua vivid-positive dead cells and GFP-negative untransfected cells. The binding of (**b**) anti-Env 2G12 control mAb or (**c**) sCD4 was detected on live GFP+ Env-expressing cells using Alexa Fluor 647-coupled secondary antibodies. (**b-c**) Shown are graphs depicting one representative replicate showing the binding of 2G12 and sCD4 on $Env_{JR\text{-}FL}$ WT or its S375H mutant in presence or absence or temsavir (TMR; 10 μM). Mean fluorescence intensities (MFI) are highlighted in green.

Supplementary References

1. Ananthaswamy, N. et al. A sequestered fusion peptide in the structure of an HIV-1 transmitted founder envelope trimer. *Nat Commun* **10**, 873 (2019).
