# Supplementary material for: Structure-function analyses reveal key molecular determinants of HIV-1 CRF01_AE resistance to the entry inhibitor temsavir
Source: Nat Commun. 2023 Oct 23;14:6710. doi: 10.1038/s41467-023-42500-2 (PMC10593844; doi:10.1038/s41467-023-42500-2)
Supplement: Supplementary file 3 — Reporting Summary [file 41467_2023_42500_MOESM3_ESM.pdf]

nature portfolio

Corresponding author(s): Marzena Pazgier

Last updated by author(s): Sep 21, 2023



# Reporting Summary

Nature Portfolio wishes to improve the reproducibility of the work that we publish. This form provides structure for consistency and transparency in reporting. For further information on Nature Portfolio policies, see our Editorial Policies and the Editorial Policy Checklist.

## Statistics

For all statistical analyses, confirm that the following items are present in the figure legend, table legend, main text, or Methods section.

| n/a | Confirmed | |
|---|---|---|
| ☐ | ☒ | The exact sample size (*n*) for each experimental group/condition, given as a discrete number and unit of measurement |
| ☐ | ☒ | A statement on whether measurements were taken from distinct samples or whether the same sample was measured repeatedly |
| ☐ | ☒ | The statistical test(s) used AND whether they are one- or two-sided<br>*Only common tests should be described solely by name; describe more complex techniques in the Methods section.* |
| ☒ | ☐ | A description of all covariates tested |
| ☐ | ☒ | A description of any assumptions or corrections, such as tests of normality and adjustment for multiple comparisons |
| ☐ | ☒ | A full description of the statistical parameters including central tendency (e.g. means) or other basic estimates (e.g. regression coefficient) AND variation (e.g. standard deviation) or associated estimates of uncertainty (e.g. confidence intervals) |
| ☐ | ☒ | For null hypothesis testing, the test statistic (e.g. *F*, *t*, *r*) with confidence intervals, effect sizes, degrees of freedom and *P* value noted<br>*Give P values as exact values whenever suitable.* |
| ☒ | ☐ | For Bayesian analysis, information on the choice of priors and Markov chain Monte Carlo settings |
| ☒ | ☐ | For hierarchical and complex designs, identification of the appropriate level for tests and full reporting of outcomes |
| ☒ | ☐ | Estimates of effect sizes (e.g. Cohen's *d*, Pearson's *r*), indicating how they were calculated |

*Our web collection on statistics for biologists contains articles on many of the points above.*

## Software and code

Policy information about availability of computer code

| | |
|---|---|
| Data collection | CryoEM data was collected with ThermoFisher EPU software v3.0 or SerialEM v4.0.27. |
| Data analysis | CryoEM data was processed or analyzed with chimeraX v1.2, UCSF chimera v1.6.2, phenix v1.20.1, relion v4.0.1, ccp-em v1.4.1, coot v0.8.9, cryosparc v4.3.0, pymol v2.5.4, or cistem v1.0. Flow cytometry was analyzed using FlowJo v10.5.3. Statistics were calculated with graphpad prism v9.4.1. |

For manuscripts utilizing custom algorithms or software that are central to the research but not yet described in published literature, software must be made available to editors and reviewers. We strongly encourage code deposition in a community repository (e.g. GitHub). See the Nature Portfolio guidelines for submitting code & software for further information.

## Data

Policy information about availability of data

All manuscripts must include a data availability statement. This statement should provide the following information, where applicable:

- Accession codes, unique identifiers, or web links for publicly available datasets
- A description of any restrictions on data availability
- For clinical datasets or third party data, please ensure that the statement adheres to our policy

Cryo-EM reconstructions and atomic models generated during this study are deposited in the Protein Data Bank (PDB) and the Electron Microscopy Data Bank (EMDB) under the following accession codes: PDB: 8DOK, 8G6U, 8CZZ and EMDB: EMD-27596, EMD-29783, EMD-27103. All data generated and reported in this

paper are available from the lead contact, Marzena Pazgier (marzena.pazgier@usuhs.edu), upon request. Structural data collection, refinement statistics and codes for deposited structures are provided in the Supplementary Information (Table S1) and source data for Figures 1, 2 and S1 are provided with this paper.

## Research involving human participants, their data, or biological material

Policy information about studies with human participants or human data. See also policy information about sex, gender (identity/presentation), and sexual orientation and race, ethnicity and racism.

| | |
|---|---|
| Reporting on sex and gender | This study does not involve human participants or human data. |
| Reporting on race, ethnicity, or other socially relevant groupings | This study does not involve human participants or human data. |
| Population characteristics | This study does not involve human participants or human data. |
| Recruitment | This study does not involve human participants or human data. |
| Ethics oversight | This study does not involve human participants or human data. |

Note that full information on the approval of the study protocol must also be provided in the manuscript.

# Field-specific reporting

Please select the one below that is the best fit for your research. If you are not sure, read the appropriate sections before making your selection.

[x] Life sciences   [ ] Behavioural & social sciences   [ ] Ecological, evolutionary & environmental sciences

For a reference copy of the document with all sections, see nature.com/documents/nr-reporting-summary-flat.pdf

# Life sciences study design

All studies must disclose on these points even when the disclosure is negative.

| | |
|---|---|
| Sample size | No sample size calculation was required. None of the methods used would require a predetermined sample size due to the nature of the study, including viral neutralization assay, sCD4 competition assay and cryo-EM structures. The viral neutralization and sCD4 competition assays were perform in three independant experiments and the reproducibility of the assays made us confident that the sample size was sufficient. |
| Data exclusions | No data were excluded from the analyses. |
| Replication | Experiments were repeated in at least three independant experiments to confirm results validity. All attempts at replication were successful. |
| Randomization | No randomization was required for this type of study. None of the methods used would require any randomization due to the nature of the study, including viral neutralization assay, sCD4 competition assay and cryo-EM structures. There is no allocation of sample groups for these assays, therefore no randomization was applied. |
| Blinding | No blinding was done for this study since no allocation of sample groups was required. All experiments were conducted unblindedly since the investigators were involved in the design, execution and analyses of the current study. |

# Reporting for specific materials, systems and methods

We require information from authors about some types of materials, experimental systems and methods used in many studies. Here, indicate whether each material, system or method listed is relevant to your study. If you are not sure if a list item applies to your research, read the appropriate section before selecting a response.

## Materials & experimental systems

| n/a | Involved in the study | |
|---|---|---|
| [ ] | [x] | Antibodies |
| [ ] | [x] | Eukaryotic cell lines |
| [x] | [ ] | Palaeontology and archaeology |
| [x] | [ ] | Animals and other organisms |
| [x] | [ ] | Clinical data |
| [x] | [ ] | Dual use research of concern |
| [x] | [ ] | Plants |

## Methods

| n/a | Involved in the study | |
|---|---|---|
| [x] | [ ] | ChIP-seq |
| [ ] | [x] | Flow cytometry |
| [x] | [ ] | MRI-based neuroimaging |

## Antibodies

| | |
|---|---|
| Antibodies used | - Monoclonal anti-Env gp120-gp41 interface 8ANC195: this antibody was produce in-house using expression plasmids kindly provided by Michel Nussenzweig (The Rockefeller University).<br><br>- Monoclonal anti-Env V3 glycan 10-1074: large-scale production of 10-1074 was conducted at Scripps Research Center for Antibody Development & Production (La Jolla, CA).<br><br>- Monoclonal anti-Env outer domain 2G12, NIH AIDS Reagent Program, Catalog#: ARP-1476<br><br>- Mouse anti-Human CD4 clone OKT4, Thermo Fisher Scientific, Catalog# 14-0048-82<br><br>- Goat anti-Human IgG (H+L) Cross-Adsorbed Secondary Antibody, Alexa Fluor 647, Thermo Fisher Scientific, Catalog# A21445<br><br>- Goat anti-Mouse IgG (H+L) Cross-Adsorbed Secondary Antibody, Alexa Fluor 647, Thermo Fisher Scientific Catalog# A21235 |
| Validation | - Monoclonal anti-Env gp120-gp41 interface 8ANC195: This antibody was first described in the following article and was shown to neutralize the HIV-1 virus (https://www.science.org/doi/10.1126/science.1207227).<br><br>- Monoclonal anti-Env V3 glycan 10-1074:This antibody was first described in the following article and was shown to neutralize the HIV-1 virus (https://www.pnas.org/doi/10.1073/pnas.1217207109).<br><br>- Monoclonal anti-Env outer domain 2G12: The manufacturer provides multiple relevant citations were this antibody neutralizes the HIV-1 virus (https://www.hivreagentprogram.org/Catalog/HRPMonoclonalAntibodies/ARP-1476.aspx).<br><br>- Mouse anti-Human CD4 clone OKT4: The manufacturer provides multiple relevant citations were this antibody was used for flow cytometry (https://www.thermofisher.com/antibody/product/CD4-Antibody-clone-OKT4-OKT-4-Monoclonal/14-0048-82).<br><br>- Goat anti-Human IgG (H+L) Cross-Adsorbed Secondary Antibody, Alexa Fluor 647: The manufacturer states that this antibody was verified by relative expression to ensure that the antibody binds to the antigen stated and provides multiple relevant citations were this antibody was used for flow cytometry (https://www.thermofisher.com/antibody/product/Goat-anti-Human-IgG-H-L-Cross-Adsorbed-Secondary-Antibody-Polyclonal/A-21445).<br><br>- Goat anti-Mouse IgG (H+L) Cross-Adsorbed Secondary Antibody, Alexa Fluor 647: The manufacturer provides multiple relevant citations were this antibody was used for flow cytometry (https://www.thermofisher.com/antibody/product/Goat-anti-Mouse-IgG-H-L-Cross-Adsorbed-Secondary-Antibody-Polyclonal/A-21235). |

## Eukaryotic cell lines

Policy information about cell lines and Sex and Gender in Research

| | |
|---|---|
| Cell line source(s) | - HEK293T, derived from the kidney of a human female embryo and modified for stable expression of the SV40 T-antigen, American Type Culture Collection (ATCC), Catalog# CRL-3216<br><br>- Cf2Th/CD4/CCR5, derived from the thymus tissue of a newborn female dog and modified for stable expression of human CD4 and human CCR5, obtained from Dr Joseph Sodroski (Dana Farber Cancer Institute).<br><br>- Expi293F GnTI-, derived from the kidney of a human female embryo lacking N-acetylglucosaminyl-transferase I (GnTI) enzyme activity and adapted for suspension culture, Thermo Fisher Scientific, Catalog# A39240<br><br>- Expi293F,derived from the kidney of a human female embryo and adapted for suspension culture, Thermo Fisher Scientific, Catalog# A14528 |
| Authentication | None of the cell lines used were authenticated. |
| Mycoplasma contamination | Cells were tested negative for mycoplasma by PCR. |
| Commonly misidentified lines<br>(See ICLAC register) | No misidentified cell lines were used in this study. |

# Flow Cytometry

## Plots

Confirm that:

☒ The axis labels state the marker and fluorochrome used (e.g. CD4-FITC).

☒ The axis scales are clearly visible. Include numbers along axes only for bottom left plot of group (a 'group' is an analysis of identical markers).

☒ All plots are contour plots with outliers or pseudocolor plots.

☒ A numerical value for number of cells or percentage (with statistics) is provided.

## Methodology

| | |
|---|---|
| Sample preparation | Two millions HEK293T cells were transfected with 7 µg of Env expressor and 1 µg of a green fluorescent protein (GFP) expressor (pIRES2-EGFP; Clontech) with the calcium phosphate method. When the pSVIII Env expressor was used, it was co-transfected with 0.25 µg of a Tat-expressing plasmid. At 48 h post-transfection, HEK293T cells were detached and washed with PBS. Cells were incubated with 10 µg/mL of soluble CD4 (sCD4) in presence of temsavir (10 µM) or an equivalent volume of the vehicle (DMSO), followed by staining performed with the monoclonal anti-CD4 OKT4 antibody (0.5 µg/mL) and goat anti-mouse IgG antibodies pre-coupled to Alexa Fluor 647 (2 µg/mL) to detect cell-bound sCD4. Alternatively, transfected HEK293T cells were stained with the anti-Env 2G12 antibody (10 µg/mL) and goat anti-human IgG antibodies pre-coupled to Alexa Fluor 647 (2 µg/mL) to detect cell-bound 2G12. Stained cells were fixed with a PBS solution containing 2% fomaldehyde. |
| Instrument | Samples were acquired on an LSR II cytometer (BD Biosciences). |
| Software | Samples were acquired using BD FACSDiva v9.0 (BD Biosciences) and data analysis was performed using FlowJo v10.5.3 (Tree Star). |
| Cell population abundance | Upon acquisition, the transfected cell population (Env+ GFP+) represented at least 10% of the live cell population. |
| Gating strategy | The gating strategy for the soluble CD4 (sCD4) competition assay is the following:<br>gate 1: FSC-A/SSC-A; to identify HEK293T cells<br>gate 2: FSC-H/FSC-W; to rule out doublet events<br>gate 3: FSC-A/Aqua vivid; to rule out dead cells (Aqua vivid negative cells)<br>gate 4: GFP/Env-Alexa Fluor 647; to quantify the binding of sCD4 or 2G12 on transfected cells (GFP positive cells) |

☒ Tick this box to confirm that a figure exemplifying the gating strategy is provided in the Supplementary Information.
